# Interrupted Inferior Vena Cava With Azygos Continuation—A Procedural Challenge During Transcatheter Aortic Valve Replacement: A Case Report

**DOI:** 10.1016/j.jscai.2026.105563

**Published:** 2026-08-04

**Authors:** Steve Kibuom Lee, KaChon Lei, Malek Al-Hawwas, William Aimar Rollefson

**Affiliations:** a Department of Cardiology, University of Arkansas for Medical Sciences, Little Rock, Arkansas; b Division of Cardiology, Scripps Clinic, La Jolla, California; c Department of Cardiology, VA Central Arkansas Health Care, Little Rock, Arkansas; d Department of Cardiology, Arkansas Heart Hospital, Little Rock, Arkansas

**Keywords:** case report, transcatheter aortic valve replacement, transvenous pacemaker, venous malformation

## Abstract

Interrupted inferior vena cava with azygos continuation is a rare congenital venous anomaly characterized by the absence of the hepatic segment of the inferior vena cava, with venous return from the lower extremities redirected through a dilated azygos vein into the superior vena cava. Although typically clinically silent, this anomaly can pose significant challenges during transcatheter procedures requiring femoral venous access. We describe a case of interrupted inferior vena cava with azygos continuation that was incidentally discovered during transcatheter aortic valve replacement after difficulty advancing a temporary transvenous pacemaker via the femoral venous approach.

## Case presentation

An 83-year-old man with a history of chronic obstructive pulmonary disease, chronic kidney disease (baseline creatinine, 1.8 mg/dL), chronic liver disease, atrial fibrillation on apixaban, chronic anemia, and longstanding lower extremity edema presented with progressive dyspnea and an episode of syncope. Laboratory evaluation revealed hemoglobin of 8.1 g/dL, creatinine of 1.8 mg/dL, and an elevated NT-proBNP level of 2740 pg/mL. Chest radiography demonstrated mild cardiomegaly with pulmonary vascular congestion.

Transthoracic echocardiography revealed severe calcific aortic stenosis with restricted leaflet motion and a left ventricular ejection fraction of 40% to 45%. Computed tomography angiography performed for transcatheter aortic valve replacement (TAVR) planning demonstrated severe valvular calcification and suitable iliofemoral arterial access. Despite extensive coronary artery calcification noted on computed tomography (CT), invasive coronary angiography showed no obstructive epicardial coronary artery disease. Given his advanced age and multiple comorbidities, the patient was referred for TAVR.

During the procedure, a temporary transvenous pacemaker was introduced via the femoral venous approach. However, the pacing lead could not be advanced into the right ventricle. Multiple attempts using a J-tipped guide wire and an XB 3.5 catheter were unsuccessful, as the guide wire repeatedly failed to enter the right ventricle (Figure 1A). Fluoroscopy revealed an unusual cephalad trajectory of the wire.

**Figure 1:**
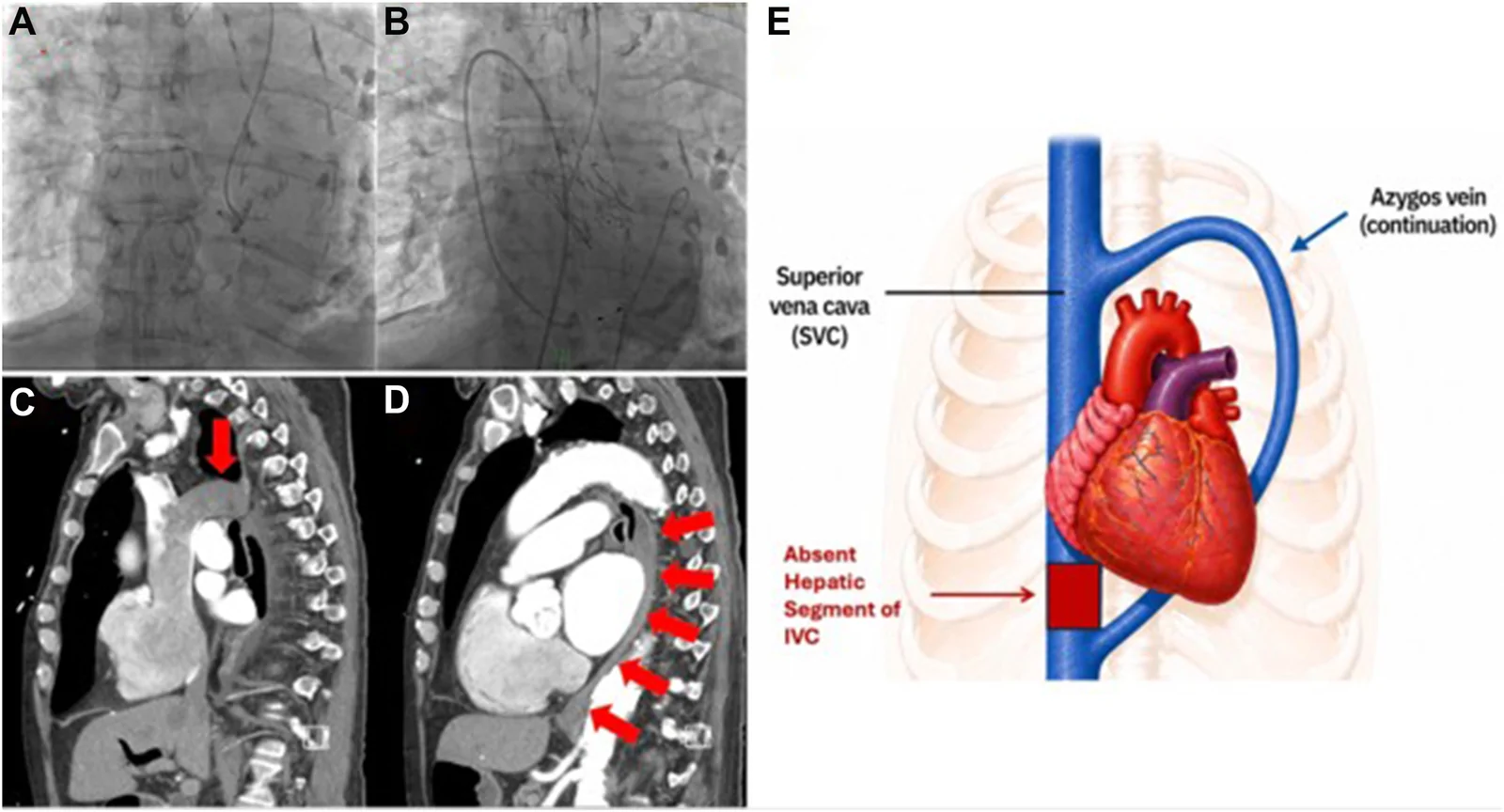
**(A) Fluoroscopy showing an XB 3.5 catheter and J-tipped guide wire from the femoral venous approach tracking cephalad without entering the right atrium or right ventricle.** (**B**) Fluoroscopy demonstrating temporary pacing lead advancement through the IVC into the azygos vein and SVC before reaching the right ventricle. (**C**) CT image demonstrates the absence of the hepatic segment of the IVC with azygos continuation draining into the superior vena cava. The arrow indicates the dilated azygos vein continuing into the SVC. (**D**) CT image showing compression of the IVC adjacent to the left atrium, potentially contributing to chronic lower extremity edema. The arrow indicates the continuation of the IVC coursing posterior to the left atrium and draining into the azygos vein. (**E**) This schematic demonstrates an interrupted IVC with azygos continuation, characterized by the absence of the hepatic segment of the IVC, resulting in venous return from the lower extremities being redirected through a dilated azygos vein into the SVC.

Given this atypical course, careful manipulation allowed further advancement of the wire, which was ultimately found to be traversing an azygos continuation. The pacemaker lead was successfully advanced through the azygos vein into the superior vena cava (SVC), then into the right atrium, and subsequently positioned in the right ventricle. Adequate pacing capture was confirmed (Figure 1B). The TAVR procedure was then completed without complications.

Postprocedural reassessment of the venous anatomy, including review of preprocedural CT imaging, demonstrated the absence of the hepatic segment of the inferior vena cava (IVC). Venous return from the lower extremities was noted to course through a markedly dilated azygos vein draining into the SVC, while the hepatic veins drained directly into the right atrium (Figure 1C). These findings were consistent with interrupted IVC with azygos continuation.

## Discussion

Interrupted IVC with azygos continuation is a rare congenital venous anomaly that results from failure of the hepatic segment of the IVC to develop during embryogenesis (Figure 1E). In this condition, venous return from the lower body bypasses the absent hepatic segment and is redirected into the azygos venous system, which then drains into the SVC. The hepatic veins typically drain directly into the right atrium. The reported prevalence is approximately 0.6% to 2% among patients with congenital heart disease and roughly 0.3% in the general population.^1^^,^^2^

Most patients with this anomaly remain asymptomatic, and the condition is frequently discovered incidentally during imaging studies or invasive procedures.^2^, ^3^, ^4^, ^5^ However, its presence may have important implications for structural heart and electrophysiologic interventions that rely on femoral venous access. The elongated and tortuous course through the azygos vein can make catheter advancement challenging and may prevent devices from following the expected trajectory into the right atrium.

In procedures such as TAVR, temporary transvenous pacing is commonly performed via the femoral vein because it allows rapid access and stable positioning during valve deployment. When an interrupted IVC is present, the pacing lead must traverse the azygos vein before reaching the SVC and right atrium. This circuitous pathway may result in unusual wire trajectories, difficulty advancing catheters, and prolonged procedural time. Repeated attempts at catheter manipulation without recognition of the underlying venous anatomy may increase the risk of venous injury or cardiac perforation.

Recognition of atypical wire movement during fluoroscopy is therefore essential. In our case, the persistent cephalad trajectory of the wire without entry into the right ventricle prompted reconsideration of the venous anatomy, leading to identification of an interrupted IVC with azygos continuation. This recognition enabled an appropriate adjustment of the procedural strategy and the successful completion of the intervention.

Preprocedural CT imaging is routinely obtained for TAVR planning, primarily to evaluate aortic annular measurements and iliofemoral arterial access. However, these scans also provide valuable information regarding venous anatomy. A systematic review of venous structures may help identify anomalies, such as an interrupted IVC, prior to the procedure.^3^ In our patient, preprocedural imaging also demonstrated compression of the IVC adjacent to the left atrium, which may have contributed to impaired venous return and the patient’s longstanding lower extremity edema (Figure 1D). Prior studies have shown that symptomatic patients with interrupted IVC may present with deep vein thrombosis, leg swelling, leg pain, and lower extremity varices, consistent with our patient’s clinical findings.^4^

Recognition of such findings is critical for procedural planning and may help avoid unexpected technical challenges during femoral venous access. In such cases, alternative strategies—internal jugular venous access or left ventricular guide wire pacing—should be considered when femoral venous pacing is not feasible.

This case highlights several important procedural lessons. First, atypical wire or catheter trajectories during femoral venous access should prompt immediate consideration of underlying venous anomalies. Second, preprocedural CT imaging obtained for arterial access planning should also be carefully reviewed for venous anatomy. Finally, familiarity with alternative venous access strategies or modified wiring techniques may help prevent procedural delays and technical failure. For structural heart operators, early recognition of anomalous venous anatomy is essential to facilitate procedural planning and minimize unnecessary catheter manipulation and potential complications.

## Conclusion

Interrupted IVC with azygos continuation can significantly complicate femoral venous access during TAVR. Careful review of preprocedural imaging and heightened awareness of venous anomalies are essential for procedural planning and successful device delivery.

## CRediT authorship contribution statement

**Steve Kibuom Lee:** Writing – review & editing, Writing – original draft, Formal analysis, Data curation, Conceptualization. **KaChon Lei:** Writing – review & editing, Writing – original draft, Conceptualization. **Malek Al-Hawwas:** Writing – review & editing, Writing – original draft, Formal analysis, Data curation, Conceptualization. **William Aimar Rollefson:** Writing – review & editing, Writing – original draft, Data curation, Conceptualization.

## Declaration of competing interest

The authors declared no potential conflicts of interest with respect to the research, authorship, and/or publication of this article.

## References

[1] Vijayvergiya R., Bhat M.N., Kumar R.M., Vivekanand S.G., Grover A. (2005). Azygos continuation of interrupted inferior vena cava in association with sick sinus syndrome. Heart.

[2] Ghandour A., Partovi S., Karuppasamy K., Rajiah P. (2016). Congenital anomalies of the IVC-embryological perspective and clinical relevance. Cardiovasc Diagn Ther.

[3] Knol W.G., Oei F.B., Budde R.P.J., Ter Horst M. (2021). A case report of an interrupted inferior vena cava and azygos continuation: implications for preoperative screening in minimally invasive cardiac surgery. Eur Heart J Case Rep.

[4] Koc Z., Oguzkurt L. (2007). Interruption or congenital stenosis of the inferior vena cava: prevalence, imaging, and clinical findings. Eur J Radiol.

[5] Saliba T., Rotzinger D., Fahrni G. (2026). Congenital absence of the inferior vena cava with azygos continuation. Radiol Case Rep.

